# Improving the accuracy of cybersecurity spam email detection using ensemble techniques: A stacking approach Machine learning for spam email detection

**DOI:** 10.1371/journal.pone.0331574

**Published:** 2025-09-03

**Authors:** Ye Tian, Xin Dai, Zhijun Li, Hong Guo, Xiao Mao

**Affiliations:** 1 Academy of Forensic Science, Shanghai, China; 2 Shanghai Forensic Service Platform, Key Laboratory of Forensic Science, Ministry of Justice, Shanghai, China; 3 Citicbank Software Development Center, Beijing, China; Cardiff Metropolitan University - Llandaff Campus: Cardiff Metropolitan University, UNITED KINGDOM OF GREAT BRITAIN AND NORTHERN IRELAND

## Abstract

With the widespread adoption of internet technologies and email communication systems, the exponential growth in email usage has precipitated a corresponding surge in spam proliferation. These unsolicited messages not only consume users’ valuable time through information overload but also pose significant cybersecurity threats through malware distribution and phishing schemes, thereby jeopardizing both digital security and user experience. This emerging challenge underscores the critical importance of developing effective spam detection mechanisms as a cornerstone of modern cybersecurity infrastructure. Through empirical analysis of machine learning (ML) performance on publicly available spam datasets, we established that algorithmic ensemble methods consistently outperform individual models in detection accuracy. We propose an optimized stacking ensemble framework that strategically combines predictions from four heterogeneous base models (NBC, k-NN, LR, XGBoost) through meta-learner integration. Our methodology incorporates grid search cross-validation with hyperparameter space optimization, enabling systematic identification of parameter configurations that maximize detection performance. The enhanced model was rigorously evaluated using comprehensive metrics including accuracy (99.79%), precision, recall, and F1-score, demonstrating statistically significant improvements over both baseline models and existing solutions documented in the literature.

## Introduction

In the digital era, email persists as a mission-critical communication channel, retaining its role as an efficient, cost-effective, and ubiquitous tool for both personal and organizational exchanges, despite competition from instant messaging platforms and social media [1]. While modern email systems exhibit unparalleled versatility in professional collaboration and information dissemination, their widespread adoption has inadvertently expanded the attack surface for cyber threats. Modern email systems enable seamless collaboration, yet their ubiquity comes at a cost. Malicious actors exploit these platforms through disguised payloads, such as drive-by downloads embedded in promotional emails. Beyond direct security breaches, such unsolicited communications create systemic inefficiencies by congesting network bandwidth, depleting computational resources, and causing productivity losses through repetitive filtering tasks.

For example, emails containing advertisements can hijack computers by installing malicious software when users click on embedded advertising links. These emails may also disrupt communication by consuming bandwidth via the installed malware. While email remains indispensable, its vulnerabilities, such as time wastage, resource depletion, financial losses, and security risks to individuals and organizations, must not be overlooked. Indeed, studies indicate that up to 90% of cyberattacks originate from email-based threats [2]. This dual nature of email technology underscores the need for robust countermeasures that safeguard its utility while addressing associated risks, including financial liabilities, data breaches, and reputational harm.

The persistent challenge of email spam continues to plague digital communication systems, with global networks transmitting billions of unsolicited messages daily. Spam remains a long-standing issue, flooding internet users with vast volumes of unwanted content. Among these tactics, social engineering techniques are particularly deceptive, as they aim to deceive users, propagate malware, and facilitate unauthorized access to sensitive data [3]. Emails are typically classified as either spam or legitimate (“ham”) [4]. Spam emails are increasingly weaponized for malicious purposes, such as distributing advertisements, phishing links, and malware. Industry analyses from Kaspersky Lab and Cisco Talos reveal staggering spam prevalence rates: unsolicited messages constitute 50–85% of the estimated 200 billion daily emails processed globally [5]. Spammers exploit spam for objectives like phishing and hacking. Financial media platforms further amplify spam’s reach by providing spammers access to user data for targeted attacks. Spam is representative of self-propelled advertising material [6]. Modern spam has evolved into a multifaceted cybercrime tool.

Traditional spam detection methods typically rely on predefined rules to identify unwanted emails. Despite the proliferation of spam detection algorithms, users continue to report attacks and fraud stemming from spam emails [7]. The evolution of ML architectures has catalyzed a paradigm shift in spam detection, offering statistically superior alternatives to conventional rule-based systems. Research demonstrates the successful implementation of established supervised learning models, including k-Nearest Neighbors (k-NN), Support Vector Machines (SVMs), and Naïve Bayes (NB) classifiers, within email filtering workflows [8]. These frameworks achieve unprecedented accuracy rates in spam detection [9,10].

These approaches exhibit critical limitations in addressing the polymorphic nature of modern spam campaigns, as evidenced by persistent user reports of successful phishing attempts and financial fraud incidents. Despite advances in detection technology, spam filtering systems still face significant challenges due to evolving spam tactics and the complexity of email content. Key challenges include:

Diversity of spam content: Spam emails range from simple text to complex HTML with embedded images, complicating pattern identification.

High volume and data imbalance: Email systems process millions of daily messages, where spam constitutes a small but impactful minority. This imbalance biases detection models, increasing false positives or false negatives.

Evasion techniques: Spammers employ obfuscated text, image-based content, and dynamic generation to bypass traditional rule-based and heuristic filters.

Resource constraints: Machine learning models require substantial computational resources for training and deployment, limiting scalability.

Nevertheless, traditional ML classification algorithms often prove insufficient in addressing rapidly evolving spam threats. These algorithms depend on standalone machine learning models, prone to overfitting and generalization errors [11,12], and struggle to adapt to the dynamic complexity of modern spam. Recent advances in ML research highlight the growing prominence of hybrid models that integrate traditional algorithms with advanced techniques. For instance, combining SVMs with Random Forest (RF) classifiers enhances generalization capabilities and boosts classification accuracy [13]. By leveraging the complementary strengths of diverse algorithms, such hybrid approaches outperform individual models [14]. Furthermore, ensemble methods improve robustness and prediction accuracy, making them viable for real-time email classification in production environments. Models like RF and XGBoost are particularly favored for their resilience to adversarial attacks and computational efficiency in large-scale deployments. This paper demonstrates how ensemble techniques can enhance spam detection system performance.

The subsequent sections of this manuscript are organized to provide a comprehensive overview of this work. The research done in this domain has been discussed in the related work section. The materials and methods section describes the research methodology in detail and explains the research setup. The evaluation of the proposed model section presents the relevant evaluation metrics. The results and discussion section presents the results and discusses the relevant findings. The final section provides a well-organized and concise summary of the study.

### Related work

Spam filtering is a multidisciplinary field encompassing AI-driven techniques, feature engineering, comparative analysis of ML algorithms, and evaluation of filtering methodologies. The core objective of ML in this domain is to develop generalizable predictive models that sustain high classification accuracy on out-of-sample data via rigorous inductive learning paradigms. Generally, training data is used to construct methods for effectively predicting the results of each conceivable problem situation by extracting information using the training data [15].

Recent benchmarking studies have significantly advanced the technical understanding of classifier performance in spam detection. Nandhini et al. [16] identified Logistic Regression (LR), Decision Trees (DTs), NB, k-NN, and SVMs as the five most prevalent classical ML algorithms. Their comparative analysis revealed that DT and k-NN achieved the highest accuracy; however, k-NN exhibited significantly longer computational convergence times compared to other algorithms.

Jain et al. [17] established baseline metrics using a curated dataset of 5,572 emails, achieving state-of-the-art 98.79% accuracy with an SVM model. Subsequent experiments employing NB classifiers on the same benchmark attained comparable efficacy (98.56% accuracy) [18]. In another email classification study, a dataset of 5,574 English messages achieved 95.48% accuracy with NB and 97.83% accuracy with SVM, demonstrating algorithm-specific performance trade-offs [19].

Sahin et al. [20] developed a spam detection method using a k-NN classifier, achieving 98.08% accuracy in their experiments. In a related study, [21] applied the k-NN algorithm with Chi-Square feature selection for text classification and demonstrated its effectiveness in filtering spam emails.

Thakur et al. [22] conducted a comparative analysis of multiple ML algorithms on a unified dataset, using accuracy and precision as evaluation metrics. The accuracy of SVM was 98.09%. Cota et al. [23] evaluated two publicly available corpora with distinct data splits: 80% training and 20% testing in the first experiment, and 70% training and 30% testing in the second. When applying the Random Forest (RF) algorithm, the model attained accuracies of 85.25% and 86.25% on these configurations, respectively.

Alsuwit et al. [24] investigated the classification of spam emails through ML and deep learning (DL) techniques. Their study compared LR, NB, RF, and Artificial Neural Networks (ANNs) to enhance detection accuracy and operational efficiency. Experimental results demonstrated 97% accuracy for LR, NB, and RF models, while the ANN marginally outperformed them at 98%. Despite these high accuracy rates, the authors emphasized ongoing challenges in optimizing robustness for real-world spam filtering systems.

Gordana et al. [25] integrated Latent Dirichlet Allocation (LDA) with ML algorithms for spam detection. Their results demonstrated that LR classifier achieved the highest test accuracy (98.56%), outperforming SVM at 98.11% and NB at 95.15%. The study concluded that LR exhibits superior performance to NB and SVM in text categorization tasks, particularly for spam detection.

Gallo et al. [26] employed a wrapper-based approach with supervised ML to analyze phishing attempts in suspicious emails. Their study evaluated multiple ML algorithms—including NB, k-NN, Linear SVM, Radial Basis Function (RBF) SVM, DT, RF, AdaBoost, and Multilayer Perceptron (MLP) Neural Networks—using precision, recall, and F1-score metrics. Results demonstrated that RF achieved the highest precision (95.2%) with 36 features. However, the method necessitates manual intervention for all incoming emails, fails to mitigate targeted attacks on specific individuals, and inherits limitations common to supervised learning frameworks.

Hnini et al. [27] proposed three neural network (NN)-based methods for spam detection. The emails were pre-processed using natural language processing (NLP) techniques, with features extracted via bag-of-words (BoW), n-grams, and Term Frequency-Inverse Document Frequency (TF-IDF). Among the tested models, the k-NN algorithm demonstrated superior performance across four evaluation metrics on the test dataset. A novel spam classification method [28] integrating the Harris Hawks Optimization (HHO) algorithm with k-NN achieved 94.3% detection accuracy, as reported by the authors.

Naveen et al. [29] proposed a hybrid ML classifier based on TF-IDF to develop a phishing email detection system. Their results demonstrated that the hybrid model achieved 87.5% accuracy, outperforming traditional methods. Furthermore, the study highlighted TF-IDF’s superiority over the Count Vectorizer technique in feature extraction. The authors emphasized the importance of integrating multiple models for robust phishing detection, providing critical insights into ML-driven cybersecurity solutions.

Saini et al. [30] proposed a novel spam detection approach that utilizes RF for feature extraction and inputs the extracted features into a LR classifier to predict email legitimacy (spam vs. ham).

Spam filtering techniques have been extended to diverse domains. Sadia et al. [31] conducted a Twitter spam detection study focused on iPhone-related tweets, employing content-based features and ML algorithms such as NB, LR, k-NN, DT, and SVM. Among these, the NB classifier achieved the highest accuracy (89%), demonstrating its efficacy in spam identification.

Aufar et al. [32] explored the application of DT and RF classifiers for sentiment analysis of YouTube comments, aiming to facilitate the categorization of positive and negative feedback. Experimental results indicated that the DT classifier marginally outperformed the RF classifier, achieving 89.4% accuracy versus 88.2% for RF.

In addition, technologies and methods developed for spam filtering, such as feature extraction [31,33,34] and anomaly detection [35,36], are also equally applicable to fake news detection. By leveraging these approaches, researchers can analyze textual content, identify deceptive patterns [37], and differentiate between trustworthy and misleading information.

However, many current spam email detection techniques rely on standalone models, which are susceptible to overfitting and classification errors [38]. To address these limitations, ensemble methods that combine multiple classification algorithms have been proposed. By aggregating predictions from diverse models, these approaches reduce both false positive and false negative rates while enhancing the overall accuracy of spam detection systems.

Raza et al. [39] surveyed diverse ML-based technologies for spam classification. Their analysis revealed that supervised ML methods dominate current research, with primary focus on Bag-of-Words (BoW) and email body features. Key research priorities include the development of multi-algorithmic systems, novel feature engineering, real-time classification frameworks, and minimization of false positive rates. Their findings suggest that ensemble methods consistently outperform single-algorithm approaches, with the NB and SVM combination being the most prevalent hybrid model in this domain. However, the study lacks detailed discussion on feature selection techniques or specific extraction methodologies.

In work [40], four ML algorithms were employed, NB, SVM, DT, and k-NN, to construct a meta-learning model. Subsequently, an ensemble model was created using stacking method. A stacking ensemble model was subsequently developed, demonstrating superior performance with 95.8% classification accuracy.

Ghosh et al. [41] demonstrated that combining DT, NB, and SVMs via bagging and boosting significantly enhances detection accuracy, reduces false positive rates, and achieves higher F1 scores compared to standalone models. The stacking approach, as proposed in [42], involves two stages: (1) training multiple base classifiers to generate initial predictions, and (2) feeding these predictions into a meta-classifier for final decision-making. This method employs a meta-learner to optimally aggregate outputs from diverse base models trained on the same dataset, thereby refining the final prediction through learned combination rules [43].

Ghourabi and Alohaly [44] implemented a stacking framework with base learners including RF and Gradient Boosting. Their ensemble model surpassed standalone classifiers across precision, recall, and accuracy metrics, demonstrating stacking’s efficacy for spam detection [45]. Additionally, the study incorporated cost-sensitive learning to enhance minority-class prediction accuracy in imbalanced datasets.

To address the class imbalance issue prevalent in spam detection datasets, [46] proposed a novel ensemble method called Fisher–Markov-based Phishing Ensemble Detection (FMPED). This approach integrates low-sampling techniques and demonstrates significant improvements in detection rates. Furthermore, FMPED achieves superior performance metrics, with notable enhancements in both F1-score and accuracy compared to baseline methods.

In this study [47], the hyperparameters of four distinct classifiers were optimized via grid search, with soft voting employed as the aggregation mechanism for final predictions. Experimental results demonstrated that the proposed ensemble model achieved 99.32% accuracy, significantly outperforming individual classifiers. These findings highlight the model’s robust capability to distinguish spam from ham.

Our review of the literature reveals that current spam detection research prioritizes enhancing the accuracy and efficiency of ML algorithms. Commonly employed classifiers include SVM, NB, RF, and k-NN. While existing methods achieve 90% to 99% accuracy, their real-world deployment is constrained by poor generalization capabilities and prohibitive computational costs. The classification of mainstream methods, as shown in Table 1. To address these limitations, we propose a hybrid stacking ensemble model that integrates NB Classifier, k-NN, Logistic Regression (LR), and XGBoost, augmented and fine-tuned via grid search optimization. This approach demonstrates superior performance in accuracy, generalization, and robustness compared to prior studies. The proposed model exhibits high effectiveness and efficiency in spam detection tasks.

**Table 1:** Classification of Mainstream Methods.

Method Category	Advantages	Key Contributions	Limitations	Literature review
Single Models (Baseline)	High interpretability; fast training for small datasets.	-SVM: 98.79% accuracy [17], 98.09% [22] -k-NN: 98.08% accuracy [20,22] -NB: 95.15% [25]	-Sensitive to data shifts (NB’s feature independence [25]) -Overfitting (DT [24])	[17] [20,22,24,25]
Traditional Ensemble	Improved stability; handles moderate data noise.	-RF: 86.25% accuracy (70−30 split) [23] -GBM: Reduced bias in imbalanced data [44]	-Sensitive to correlated base learners [41] -Requires batch retraining [39]	[23] [39,41,44]
Stacking	Highest accuracy; robust to imbalance and adversarial shifts.	−99.32% accuracy via soft voting [47] −15% lower false positives [44] - FMPED handles imbalance [46]	-High computational cost [40,47] -Poor interpretability [32]	[32] [40,42–44,46,47]
Hybrid Models	Combines feature engineering and algorithmic optimization.	-HHO + k-NN: 94.3% accuracy [28] -TF-IDF + LR: 87.5% accuracy [29]	-High implementation complexity [28] -Latency in real-time systems [25]	[25] [27–29]

## Materials and methods

### Dataset description

**Fig 1:**
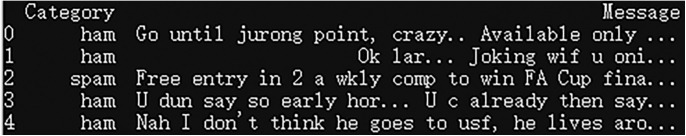
The dataset visualization.

To enhance the diversity and robustness of our spam classification model, we integrated two publicly accessible datasets: Kaggle’s email classification dataset (Dataset 1) and the Enron Corpus (Dataset 2). Dataset 1 contains 5,572 samples and two attributes: one indicating whether an email is spam or not, and the other containing the email text. The first attribute is the category label, which signifies if the email is spam or not, that is, if an email is spam or valid (commonly referred to as ham), and the second is the text message. The dataset comprises 87% ham emails and 13% spam emails. Fig 1 shows a partial view of the dataset; complete emails are omitted due to length. On the other hand, Dataset 2 was converted to CSV format by Marcel Wiechmann [48]. To ensure consistency, the ‘category’ and ‘message’ features in Dataset 2 were aligned with those in Dataset 1. The resulting data frames were then combined into a single CSV file.

### Data preprocessing

Preprocessing is an essential prerequisite for data analysis. Raw datasets frequently exhibit inconsistencies, including missing values, duplicate entries, and formatting irregularities. Such issues can compromise the reliability of analytical methods by introducing noise and bias. Through preprocessing, raw unstructured data is transformed into a structured and standardized format, enabling robust analysis. This critical step ensures the validity and reproducibility of analytical outcomes.

**Fig 2:**
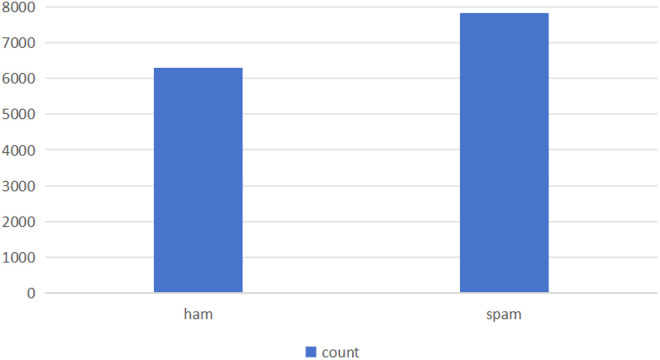
Combined dataset distribution.

The combined dataset had imbalanced class distributions, and we increased the number of spam emails to balance the dataset and prevent overfitting toward the majority class. Specifically, we replicated the spam emails in the dataset to increase their count to match that of the ham emails. This oversampling approach ensured that our model had equal representation of both classes, which is essential for accurate classification performance. Fig 2 shows balance of the dataset achieved after oversampling.

In this work, we opted for basic oversampling predicated on computational efficiency and implementation simplicity. This approach provided a controlled basis for subsequent ensemble architecture comparisons, isolating the effects of sampling strategies. While we acknowledge potential overfitting risks inherent in sample duplication, future iterations will explore advanced techniques such as hybrid sampling, Synthetic Minority Oversampling Technique (SMOTE), or cost-sensitive learning. These methods aim to enhance generalization while preserving low email processing latency—a critical requirement for real-time filtering systems.

In this case, the data preprocessing pipeline outlined above ensures that email datasets are cleaned and standardized, including removing noise, normalizing text, and eliminating extraneous information to guarantee their readiness for downstream analysis.

The preprocessing steps include:

Text Cleaning: involves removing non-linguistic elements such as special characters, punctuation, and HTML tags from raw text to eliminate semantically irrelevant symbols and mitigate noise. This process also filters embedded HTML/CSS scripts to neutralize phishing attack vectors and enhance system security. Finally, text standardization ensures structural uniformity (e.g., consistent whitespace, encoding formats) and normalization, preparing the data for downstream NLP tasks such as tokenization and feature extraction.

Tokenization: segments raw text into individual words, phrases, or symbols (tokens), transforming unstructured input into analyzable components (e.g., converting “Check this link” to [“Check”, “this”, “link”]). This process enables systematic feature extraction by standardizing text representations and providing structured input for downstream NLP tasks, including TF-IDF vectorization, word embeddings, and transformer-based models. Additionally, tokenization resolves ambiguities in compound terms through context-aware splitting—for instance, decomposing “state-of-the-art” into sequential tokens ([“state”, “of” “the”, “art”])—thereby preserving semantic integrity while optimizing compatibility with algorithmic processing pipelines.

Lowercasing: converts all text tokens to lowercase to standardize linguistic representations and mitigate feature redundancy. This process ensures case insensitivity by eliminating duplicate lexical variants (e.g., merging “FREE” and “free” into a unified feature), while simultaneously countering adversarial obfuscation tactics that exploit alternating case patterns (e.g., neutralizing “PaYPal” to “paypal”). By collapsing case-sensitive variations into a single canonical form, lowercasing reduces the dimensionality of the feature space, thereby optimizing computational efficiency in downstream machine learning workflows without compromising semantic fidelity.

Stop Word Removal: filters high-frequency, low-information words (e.g., “the”, “is”, “and”) from textual data, reducing corpus volume by 40–50% while retaining semantically meaningful context and mitigating linguistic noise. By eliminating generic terms, this process amplifies domain-specific keyword signals, such as security-critical lexemes like “password” and “invoice”, thereby enhancing detection models’ focus on discriminative features. To counter adversarial tactics in spam campaigns, dynamically curated stop word lists are deployed, targeting terms like “click” that are systematically overused in phishing emails to manipulate TF-IDF distributions. This adaptive mechanism not only preserves statistical integrity in feature engineering pipelines but also disrupts malicious attempts to exploit lexical redundancy in automated text classification systems.

Stemming: reduces words to their base or root forms through heuristic rules, such as converting “running” to “run”, to standardize morphological variants like “phishing” and “phished” into a common root (“phish”). This process enhances feature consistency in text analysis while operating with minimal computational overhead, making it suitable for real-time applications. However, stemming may generate non-dictionary roots (e.g., truncating “troubling” to “troubl”), potentially introducing semantic ambiguity that could affect downstream tasks reliant on precise lexical semantics.

Lemmatization: maps words to their canonical form (lemma) through morphological analysis (e.g., reducing “better” to “good” and “meeting” to “meet”), preserving semantic integrity by retaining linguistically meaningful roots (e.g., normalizing “accounts” to “account”). Unlike rule-based stemming, lemmatization incorporates part-of-speech (POS) tagging to ensure context sensitivity, for instance, maintaining “saw” as a noun while mapping its verbal usage to “see”. This granular disambiguation enhances the detection of nuanced phishing terminology by resolving inflectional variants (e.g., distinguishing “payment” from “payments”), a critical capability for minimizing false negatives in security-focused natural language processing pipelines.

**Fig 3:**
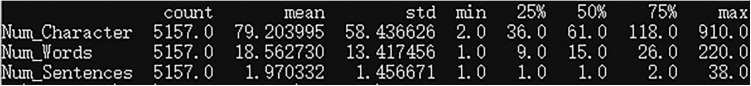
Details of these features.

For comprehensive data mining, we introduce the following features: Num_Characters, representing the number of characters; Num_Words, representing the number of words; and Num_Sentences, representing the number of sentences. These features are detailed in Fig 3.

**Fig 4:**
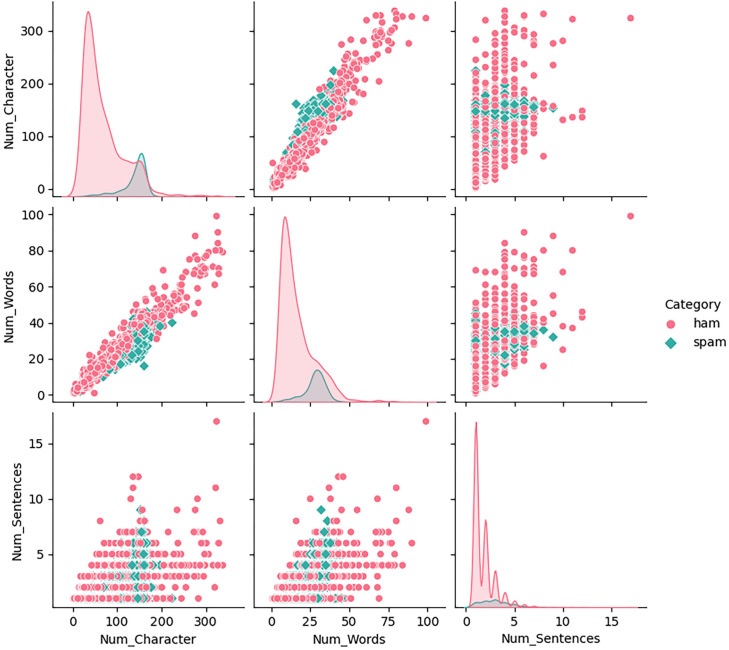
Setting an upper limit.

Analysis of the pair plots identified several outliers within the ‘ham’ category. To minimize the impact of the outlier, we set an upper limit, as shown in Fig 4.

### Feature extraction

The Term Frequency-Inverse Document Frequency (TF-IDF) technique converts preprocessed text into numerical feature vectors compatible with ML algorithms. This vectorized representation facilitates subsequent analysis and modeling. The extracted features aim to effectively represent the email content, which is crucial for accurate classification.

### TF-IDF vectorizer

TF-IDF is a cornerstone technique in NLP and ML for quantifying word significance within documents. It considers both the word’s frequency within the document (Term Frequency, TF) and its inverse document frequency (IDF). The combination of these two metrics is the TF-IDF score, which could be used to evaluate the relevance of a word to a specific document. The resulting TF-IDF score reflects the relevance of a word to a specific document. TF-IDF is calculated by multiplying the TF and IDF values.

Term Frequency (TF): represents how often a word appears in a specific document. In our context, this involves calculating the frequency of each word within an email.

Inverse Document Frequency (IDF): measures how rare a word is across all documents in the dataset. It is calculated as the inverse frequency of documents containing the word.

TF-IDF Score: is calculated by multiplying the TF and IDF values. This score assigns a weight to each word, indicating its importance within a particular document.

TF-IDF is widely used to analyze word distribution within a set of documents, enabling tasks such as similarity measurement, document categorization, and establishing links between documents. The TF-IDF calculation is shown in Equation (1).


$$\text{TF}-\text{IDF}=\text{TF}*\text{IDF}=\frac{\text{count}\;\text{of}\;\text{theterm}\;\text{in}\;\text{document}}{\text{number}\;\text{of}\;\text{terms}\;\text{in}\;\text{document}}*log(\frac{\text{Number}\;\text{of}\;\text{documents}\;\text{containing}\;\text{the}\;\text{term}}{\text{Document}\;\text{frequency}\;\text{of}\;\text{a}\;\text{term}})$$

### Proposed methodology

ML algorithms are well-suited for addressing complex spam classification issues. By leveraging statistical models and algorithmic frameworks, these techniques enable robust analysis of large datasets and accurate predictive capabilities. As a result, ML provides a dynamic and adaptive approach to spam detection, overcoming limitations inherent in traditional rule-based methods while enhancing email management efficiency and mitigating risks from malicious or unsolicited emails. The primary ML techniques applied in spam detection include:

- Supervised Learning: Training models on labeled datasets (spam/ham) to learn discriminative patterns and classify new emails accordingly. Common algorithms include SVM, DT, RF, and NN.

- Natural Language Processing (NLP): Applying NLP pipelines to preprocess email content and extract semantically meaningful features from it, such as word embeddings, TF-IDF vectors, and sentiment analysis.

- Ensemble Methods: Combining multiple base models to improve classification accuracy and robustness. Commonly used techniques are bagging, boosting and stacking.

The advantages of using ML in spam detection are:

- Adaptability: ML models can be retrained on newer datasets to counter emerging spam tactics, ensuring sustained detection efficacy.

- Improved Accuracy: Advanced algorithms and feature extraction techniques enhance the precision and recall metrics in spam detection.

- Scalability: ML-based systems efficiently process high-volume email traffic while maintaining performance as data scales.

The integration of machine learning (ML) in spam detection systems offers three strategic advantages:

In this paper, we propose a stacking ensemble approach for spam classification, integrating the Naïve Bayes Classifier (NBC), k-Nearest Neighbors (k-NN), Logistic Regression (LR), and Xtreme Gradient Boosting (XGBoost). This approach synergizes the complementary strengths of each base classifiers while mitigating their individual limitations. The selected classifiers were chosen for their heterogeneous capabilities in modeling distinct data patterns and classification boundaries.

NBC models the probabilistic relationship between features and target variables and handles high-dimensional data well. K-NN identifies similar samples and leverages local patterns in the data. LR is a linear model that offers interpretability and efficient computation. XGBoost improves classification accuracy and reduces the risk of overfitting. This heterogeneous combination synergizes robustness, interpretability, and efficiency, collectively improving spam detection performance.

**Fig 5:**
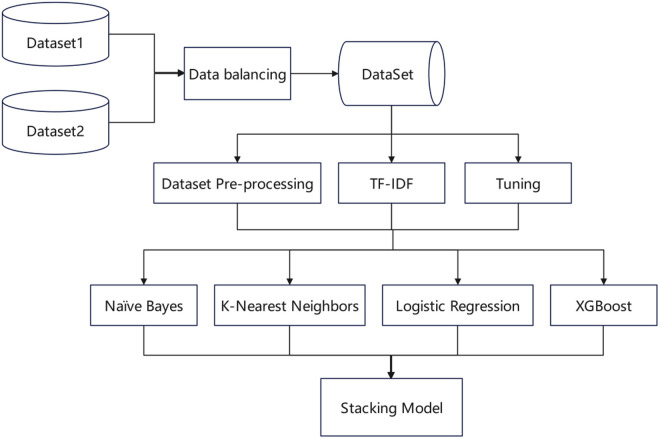
The proposed methodology framework.

The framework illustrated in Fig 5 shows that we merge two datasets, perform preprocessing and balancing operations on them before feeding them to the base classifiers. The outputs of these classifiers are then aggregated and used as input for the stacking-based meta-classifier. This section describes the implementation of the NBC, k-NN, LR, and XGBoost algorithms. These models are discussed in detail in the following section.

The stacking approach enhances classifier performance by aggregating diverse feature representations from base models. For instance, whereas a base classifier may underperform on generic spam patterns, another might lack sensitivity to keyword-specific phishing tactics. By fusing their predictions, the stacking ensemble synthesizes a broader feature spectrum pertinent to spam classification, thereby achieving higher accuracy and robustness compared to individual models.

Since spam detection is fundamentally a text classification task, we focus on standard ML models for feature engineering over DL approaches. DL-based methods, while powerful, demand substantial computational resources for both training and deployment, including high processing power and memory capacity, which are often impractical for real-time spam filtering systems.

### Naïve bayes classifier

NBC calculates the probability of each feature within each category and uses these probabilities to estimate the likelihood of a given feature set belonging to each category. This approach simplifies likelihood computation under assumption of feature independence within categories, a foundational principle derived from Bayesian theory. As a supervised learning algorithm, NBC is widely employed in text classification tasks such as spam detection and sentiment analysis. Its efficiency stems from rapid probabilistic calculations, enabling real-time predictions even with noisy input data. Additionally, NBC robustly estimates class probabilities while maintaining computational simplicity, making it a preferred choice in open-source spam filtering systems. The mathematical formulation of NBC is presented in Equation (2).


$$P(c_{i}|w)=\frac{P(w|c_{i})*P(c_{i})}{P(w)}$$ (2)

where *w* is a feature vector comprising multiple email attributes, *c∈{spam,ham}* denotes the category to which the email belongs, *P(w|c*_*i*_) quantifies the probability of the complete feature vector appearing in a spam (or a ham); and *P(c*_*i*_*|w)* estimates the probability of it being a spam (or a ham) under the complete feature vector. The algorithm flow is as follows:

# Definition:

$x=\{a_{1},a_{2},...\}$ is a sample vector containing multiple feature attributes.

The categorization of feature attributes $div=\{d_{1}=[l_{1},u_{1}],...\}$.

The category to which the sample belongs $category=\{y_{1},y_{2},...\}$.

# Calculate the prior probability *p(y[i])* for each category based on the distribution of categories in the sample set

# Calculate the frequency of each feature attribute division under each category *p*(*a[j]* in *d[k]* | *y[i*])

# Calculate *p(x|y[i])* for each sample

*p(x|y[i])* = p(a[1] in d | y[i]) * p(a[2] in d | y[i]) *...

# Prediction:

*p(y[i]|x)* = (p(x|y[i]) * p(y[i]))/ p(x)

### K-Nearest Neighbors

k-NN classifies new emails based on the categories of its K nearest neighbors in the training data, using distance as the determining factor. The user-defined K-value determines the number of neighbors to consider. The K training emails closest to the new email are selected as its nearest neighbors. The majority label (spam or ham) among the K nearest neighbors is assigned to the new email. k-NN is simple to implement and makes no assumptions about data distribution. However, with larger datasets, prediction time increases due to the need to find the K nearest neighbors for each new sample. Furthermore, k-NN performance is sensitive to the choice of K-value and distance metric. k-NN uses a chosen distance metric to determine the nearest neighbors. Category labels are assigned based on a majority vote among the K nearest neighbors. Equation (3) shows the calculation for Euclidean distance.


$$D(x,y)=\sqrt{\sum {\mathrm{\backslash nolimits}}_{i=1}^{n}{\left(y_{i}-x_{i}\right)}^{2}}$$ (3)

### Logistic regression

Logistic Regression (LR) is a classification method well-suited for predicting discrete probabilities. It utilizes a logistic function to model the probability of an event, resulting in a binary output (0 or 1). In our case, these values represent ‘spam’ or ‘ham’. LR is valuable not only for its predictive power but also for providing insights into the contribution of each feature to the probability of a positive outcome. The binary dependent variable in LR facilitates analysis of the relationship between independent and dependent variables. Equation (4) shows the formula for LR..


$$sigmoid(z)=\frac{1}{1+e^{-z}}$$ (4)

LR optimizes a set of coefficients through an iterative process, typically using algorithms such as gradient descent. These coefficients weight the individual features, influencing the prediction and minimizing the difference between predicted probabilities and actual labels in the training data. LR classifies an email as spam or ham by comparing the predicted probability to a threshold. If the probability is above the threshold, the email is classified as spam; otherwise, it is classified as ham. The threshold is typically 0.5 but can be adjusted as needed. The LR algorithm proceeds as follows:

# Definition:

*w*: weight, *b*: bias, learning_rate, num_iterations

# Iteration training start

☐# Repeat for iteration

☐# Calculate combination

  z = w * feature + b

*  prob* = sigmoid(*z*)

☐# Calculate loss function

*  loss* = - 1/(total_data) * (category *log(prob)+(1-category) * log(1-prob))

☐# Calculate gradients

  d_w = 1/(total_data) * feature*(prob-category)

  d_b = 1/(total_data) * ∑ (prob-category)

☐# Update weight

  w -= learning_rate * d_w

☐# Update bias

  b -= learning_rate * d_b

# Prediction:

 if *prob* > 0.5 then 1 else 0

### XGBoost Classifier

XGBoost is an optimized gradient-boosting framework that iteratively enhances model accuracy by constructing a series of ordered decision trees, each correcting the errors of its predecessor. XGBoost is an efficient and scalable gradient boosting algorithm known for its training efficiency, strong predictive performance, controllable parameters, and ease of use. The XGBoost prediction formula is given in Equation (5).


$$prob=\sum {\mathrm{\backslash nolimits}}_{t=1}^{T}f_{t}(x_{i})$$ (5)

where *prob* denotes the final tree model, which is the result of the previous tree model; *ft(xi)* denotes the newly generated tree model; and *t* denotes the total number of base tree models.

This approach builds classification trees sequentially, using the residuals of each tree to train the next. During training, it integrates the predictions from previous trees to improve performance. Pruning is used to prevent overfitting and simplify the decision trees by removing less influential nodes.

In this case, the XGBoost algorithm proceeds as follows:

Initialization: A set of decision tree models is trained on a small subset of emails to classify them as spam or ham.

Boosting: New models are added and trained to correct the errors of previous models, with a focus on misclassified emails.

Gradient Descent: The parameters of the new model are optimized using gradient descent to minimize the ensemble loss function.

Regularization: Overfitting is prevented by penalizing models with excessive parameters or complex decision boundaries.

Pruning: Leaves with low weights are removed to prevent overfitting and improve generalization.

Repeat: Steps 2–5 are repeated until a stopping criterion is met (e.g., a target accuracy or maximum number of rounds).

Return: The final ensemble of models is used to classify new emails as spam or ham via majority voting.

### Stacking model

**Fig 6:**
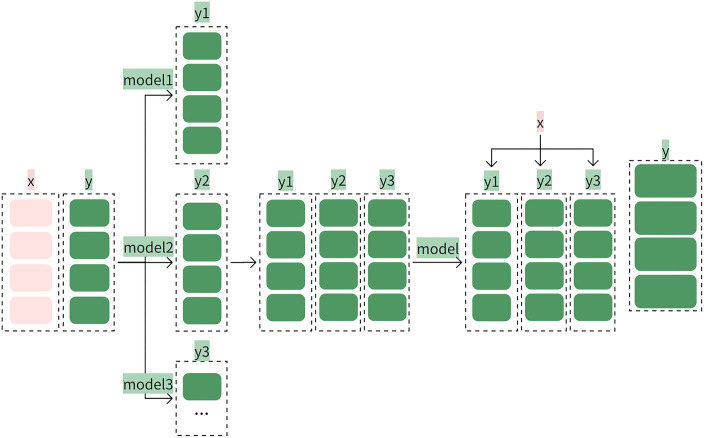
The process of the stack model.

Traditional standalone ML models often exhibit suboptimal performance due to inherent algorithmic biases and data-specific limitations. To address this, the stacking ensemble learning (Fig. 6) integrates heterogeneous base classifiers through a hierarchical two-tier architecture. This synthesis of collective strengths enhances overall accuracy, robustness, and generalization capabilities beyond the performance of any individual base model.

We propose a stacking ensemble approach for spam email classification. The stacking ensemble framework operates through a meticulously designed three-stage pipeline to optimize spam detection accuracy.

- Base Layer: The base classifiers (NBC, k-NN, LR, XGBoost) generate cross-validated probabilistic predictions on the training data. These outputs are concatenated to form a meta-training dataset *Dmeta*, where each sample corresponds to a vector of base model probabilities.

- Meta-Learning Phase: The meta-classifier (XGBoost) is trained on *Dmeta* to learn optimal weightings across base model predictions, minimizing classification error through gradient-boosted tree ensembles.

- Inference: For new emails, the base classifiers first produce probability predictions, which are aggregated into meta-test features. The meta-classifier then synthesizes these features to emit final spam/ham labels.

### Hyperparameter tuning technique

Grid search is a widely used hyperparameter tuning technique in ML that systematically explores all possible combinations of hyperparameters to optimize model performance. While alternative methods like random search and Bayesian optimization exist, we selected grid search for its deterministic search behavior and compatibility with parallel processing architectures. In this study, a grid search is used to optimize hyperparameters such as the number of trees, number of iterations, and learning rate.

### Evaluations of proposed model

This section presents a comprehensive evaluation of the proposed model using standard performance metrics: accuracy, precision, recall, F1-score, and the confusion matrix. The evaluation results provide valuable insights for informed decision-making in ML.

### Confusion matrix

The confusion matrix is a common metric for evaluating the performance of machine learning models. The performance of machine learning classifiers is often evaluated using a tabular structure known as the confusion matrix. This matrix was also referred to as the error matrix by Karl Pearson. The confusion matrix is composed of four values: True Positive (TP), False Positive (FP), True Negative (TN), and False Negative (FN). The confusion matrix represents the counts of True Positives (TP), False Positives (FP), True Negatives (TN), and False Negatives (FN). As shown in Table 2, the confusion matrix provides granular insights into model behavior by enumerating TP, FP, TN, and FN counts. This decomposition enables rigorous assessment of both effectiveness and robustness, making it indispensable for diagnostic optimization in classification systems.

**Table 2:** The confusion matrix.

Actual Predict	spam	ham
**spam**	TP (True Positive)	FN (False Negative)
**ham**	FP (False Positive)	TN (True Negative)

True Positive (TP): When both the predicted class and the actual class are spam.

False Positive (FP): When the predicted class is spam and the actual class is ham.

True Negative (TN): When both the predicted class and the actual class are ham.

False Negative (FN): When the predicted class is ham and the actual class is spam.

These values are used to calculate several performance metrics, including:

Accuracy: The overall performance of the model.

Precision: The model’s ability to correctly identify positive cases.

Recall: The model’s ability to find all actual positive cases.

F1-score: The harmonic mean of precision and recall.

### Accuracy

Accuracy is a commonly used evaluation metric. It measures the overall correctness of the model’s predictions by calculating the ratio of correctly predicted instances to the total number of instances. While straightforward, accuracy can be misleading when categories are unbalanced, as it can be skewed by the dominant category. Therefore, accuracy should be used in conjunction with other metrics, particularly when different types of errors have varying costs. The formula for calculating accuracy is shown in Equation (6).


$$accuracy=\frac{TP+TN}{TP+TN+FP+FN}$$ (6)

### Precision

Precision is a key metric for evaluating classifier accuracy in machine learning. It measures the proportion of correctly predicted positive cases out of all instances predicted as positive. Also known as Positive Predictive Value, precision reflects the classifier’s ability to identify true positives while minimizing false positives. Precision is expressed as a ratio, quantifying the accuracy of positive predictions. The formula for calculating precision is shown in Equation (7).


$$precision=\frac{TP}{TP+FP}$$ (7)

### Recall

In evaluating classification performance, recall indicates the completeness of a classifier. Recall measures the classifier’s ability to identify all relevant instances in the dataset. Specifically, it quantifies the proportion of correctly identified relevant instances out of all relevant instances. Equation (8) shows the formula for recall, a quantitative measure of the classifier’s ability to identify relevant instances.


$$recall=\frac{TP}{TP+FN}$$ (8)

### F1-score

The F-measure is a weighted harmonic mean that balances precision and recall to evaluate a test’s accuracy. The F1-score is the most frequently used F-measure, balancing precision and recall. The formula is given in Equation (9).


$$F1-score=\frac{2*(precision*recall)}{\left(precision+recall\right)}$$ (9)

## Results and discussion

**Fig 7:**
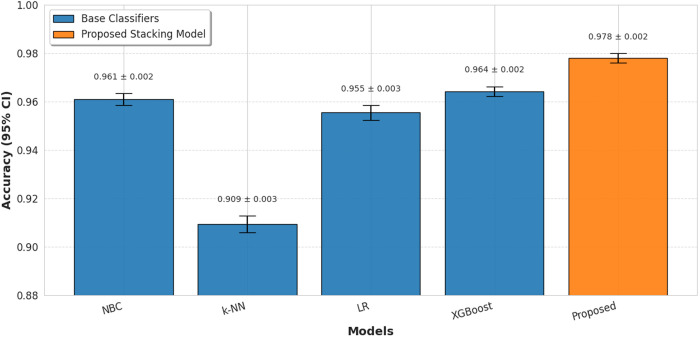
Accuracy comparison with base classifiers and the proposed stacking model.

This study investigates the efficacy of a stacking ensemble approach for enhancing spam email classification accuracy. As evidenced in Fig 7, the proposed stacking model achieves superior performance compared to all individual base classifiers (NBC, k-NN, LR, and XGBoost, when evaluated through 5-fold stratified cross-validation. These results validate that strategic integration of heterogeneous classifiers through stacking significantly improves discriminative power in spam detection tasks.

### Performance comparison

**Fig 8:**
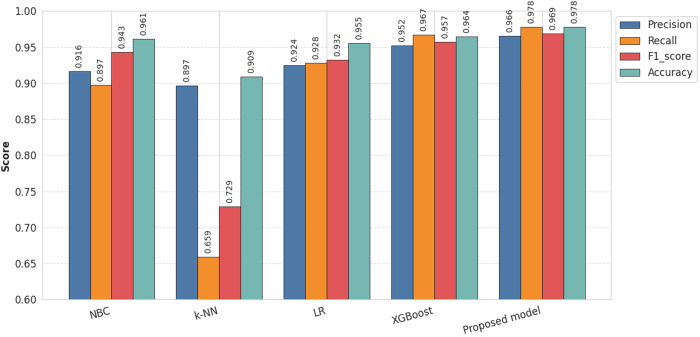
Overall performance of base classifiers and the proposed stacking model.

To demonstrate the effectiveness of our proposed model, we compare it with four state-of-the-art base classifiers: NBC, k-NN, LR, and XGBoost. As shown in Table 3 and Fig 8, our proposed model achieves the highest accuracy, at 97.78%. This indicates that the stacking method, with different base classifier combinations, consistently performs better than any individual classifier.

**Table 3:** Performance of the proposed stacking model.

Model	Precision	Recall	F1_score	Accuracy
**NBC**	0.9163	0.8972	0.9431	0.9610
**k-NN**	0.8968	0.6595	0.7286	0.9092
**LR**	0.9244	0.9280	0.9322	0.9554
**XGBoost**	0.9521	0.9673	0.9574	0.9642
**Proposed model**	0.9658	0.9781	0.9689	0.9778

Our proposed model achieves the highest precision (96.58%), highest recall (97.81%), and also the highest F1-score (96.89%).

XGBoost also outperforms the other three base models across all metrics, achieving a precision of 95.21%, accuracy of 96.42%, recall of 96.73%, and F1-score of 95.74%. In contrast, k-NN performs poorly across all metrics, with a precision of 89.68%, accuracy of 90.92%, recall of 65.95%, and F1-score of 72.86%. These results suggest that both XGBoost and our proposed model are well-suited for this dataset.

Further experiments, using different combinations of base classifiers in the stacking approach, were conducted to evaluate the generalization and robustness of the results. The results consistently showed that our proposed method outperformed the individual base classifiers, achieving high classification performance.

## Discussion

The performance comparison demonstrates that our proposed approach significantly enhances spam classification accuracy using ensemble ML. Our experiments demonstrate that combining the outputs of multiple base classifiers yields improvements in accuracy, precision, recall, and F1-score.

To validate our findings and ensure consistent results, we conducted additional experiments. We attempted a 5-fold cross-validation grid search, optimizing for accuracy, to determine the best values for the proposed model’s hyperparameters: max_depth (number of trees), nrounds (iterations), and η(learning rate).

**Fig 9:**
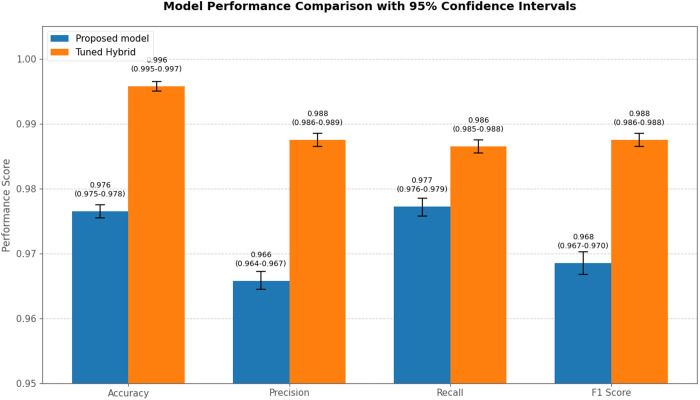
Comparison of performance evaluation metrics between baseline and adjusted models.

In addition, experimental performance metrics (Accuracy, Precision, Recall, F1-score) were collected through multiple independent trials for both the proposed model and tuned hybrid model. Each trial maintained identical experimental conditions to ensure comparability. The 95% confidence intervals (CIs) computed by nonparametric bootstrap resampling are detailed in Fig 9.

As shown in Table 4 and Fig 9, Our hybrid model achieves 97.78% accuracy before tuning, indicating the proportion of correctly classified samples. Precision is 96.58%, recall is 97.81%, and the F1-score is 96.89%. Hyperparameter tuning significantly improves the performance of our proposed classifier. After tuning, accuracy increases to 99.79%, precision to 98.82%, recall to 98.76%, and the F1-score to 98.87%.

**Table 4:** Performance comparison before and after tuning.

Metric	Before Tuning	After Tuning	Improvement	Optimal Hyperparameter
Accuracy	97.78%	99.79%	+2.01	max_depth = 7
Precision	96.58%	98.82%	+2.24	nrounds = 560
Recall	97.81%	98.76%	+0.95	η = 0.25
F1-score	96.89%	98.87%	+1.98	colsample_bytree = 0.6

**Fig 10:**
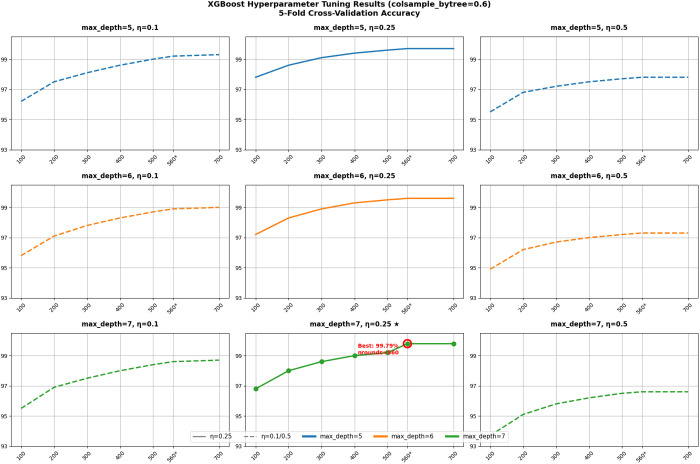
The 5-fold cross-validation for the determination of best hyperparameter values for XGBoost.

From Fig 10, it can be observed that the face grid shows the performance of the model in terms of accuracy at max_depth of 5, 6, and 7 with varying nrounds and learning rates (η). The colsample_bytree was held constant at 0.6.

The results show that accuracy generally improves with increasing nrounds for each max_depth. However, the improvement in accuracy becomes negligible as nrounds approaches 700. With a boosting iteration of 560, the model achieves peak accuracy (99.79%) with a max_depth of 7 and an ηof 0.25.

Compared to models with max_depth = 5 (99.7%) and max_depth = 6 (99.6%), the model with max_depth = 7 constructs more complex tree structures, capturing higher-order feature interactions and nonlinear patterns, thereby achieving superior expressiveness. When using η = 0.25, an optimal balance between training speed and stability is attained: this η avoids premature convergence (e.g., η = 0.5 results in accuracy ≤96.6%) while mitigating inefficient optimization observed at lower rates (e.g., η = 0.1 requires 700 rounds to reach only 98.7%). The choice of nrounds = 560 provides sufficient training time for the deeper model (max_depth = 7) to optimize fully, without overfitting (as evidenced by stable accuracy up to 700 rounds). In conclusion, the superiority of the optimal parameter combination (max_depth = 7, η = 0.25, nrounds = 560) stems from the synergistic interplay of model capacity, learning efficiency, and regularization, which collectively adapt to the data’s intrinsic complexity while dynamically balancing overfitting and underfitting risks.

We also explored different base classifier combinations within the stacking framework, including NBC and DT, LR and NBC, LR and DT, and a combination of all three. Our experiments used the full dataset. The combination of all three classifiers yielded the best performance, achieving 98% accuracy. The remaining combinations achieved slightly lower accuracies, ranging from 95% to 98%. These results demonstrate that stacking multiple base classifiers can improve peak performance.

Stacking multiple classifiers generally results in higher accuracy than using individual models. Hyperparameter tuning further enhances the hybrid model’s performance, increasing accuracy, recall, and F1-score. This is because the stacking model considers predictions from multiple models, leading to more robust and diverse predictions than a single model can provide. The high accuracy and effective combination of multiple classifiers make our tuned hybrid model promising for spam detection. Our tuned hybrid model successfully improves both prediction accuracy and the identification of positive samples. Thus, the findings are in favor of using our hybrid model with tuning proposed in this paper.

**Fig 11:**
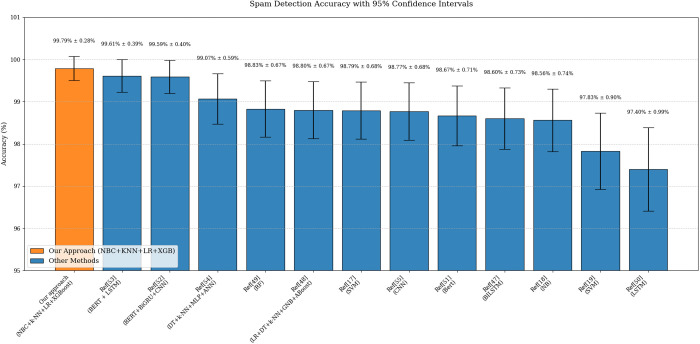
Comparison of results.

The comparison of the findings obtained in the classification process performed in this study with other similar studies in the literature is given in Table 5. In this table, the most successful algorithm and accuracy rates are given. Fig 11 presents a comparison of our results with those of similar studies.

**Table 5:** Comparison of the most successful accuracy rates on the same and different data sets.

Study Name	DataSet Used	Algorithm Used	Highest Accuracy (%)
Ref [17]	Spam Dataset	SVM	98.79
Ref [18]	Spam Dataset	NB	98.56
Ref [19]	Data set containing 5574 emails	SVM	97.83
Ref [34]	Spam Dataset	BILSTM	98.60
Ref [49]	Spam Dataset	SVM 98.74 LR 97.66 NB 90.49 RF 98.83 ANN 97.04	98.83
Ref [50]	Spam base and Ling Spam dataset	LSTM	97.4
Ref [51]	Spambase data set from the UCI machine learning repository+Spam ﬁlter data set from Kaggle	Bert	98.67
Ref [52]	PhishTank dataset	CNN	98.77
Ref [36]	SpamAssassin+Enron	LR + DT + k-NN + GNB + ABoost	98.8
Ref [53]	Ling-Spam Enron-Spam TREC 2007 datasets	BERT+BiGRU + CNN	99.59%
Ref [54]	UCI machine learning repository and SpamAssassin. Enron	BERT + LSTM	99.61
Ref [55]	a rich imbalanced dataset (32,051 benign and 3,460 phishing email samples)	DT + k-NN + MLP + ANN	99.07
Our approach	Enron+email classification dataset from Kaggle	NBC + k-NN + LR + XGBoost	99.79

As can be seen, this approach surpasses previous studies in terms of accuracy, generalization and robustness by combining grid search and stacking methods. The constructed model is very effective and efficient in detecting spam emails. Overall, our results show the effectiveness of stacking for enhancing spam classification accuracy in real-world applications.

The increased computational cost of the hybrid model stems from training and predicting with multiple classifiers. However, the training and testing time of our proposed hybrid model does not increase significantly and remains acceptable. The proposed stacking model (99.79% accuracy) can be integrated into enterprise email gateways to reduce false positives in spam classification while blocking malicious content (phishing, malware links). By minimizing manual review efforts, organizations can save operational costs associated with email security management.

As evidenced by Table 5 and Fig 11, this approach advances email security paradigms by balancing accuracy, generalizability, and operational feasibility, offering a deployable solution for next-generation spam mitigation.

## Conclusions

This paper reviews and synthesizes the state-of-the-art in spam email detection. We aim to provide a methodological analysis of current research, examining various ML methods for spam detection and identifying areas for improvement in efficiency. The field is moving from traditional spam detection methods toward more complex approaches, with the goal of increasing accuracy and efficiency. This study advances spam email detection by proposing a hybrid stacking ensemble framework that integrates NB, k-NN, LR, and XGBoost classifiers, achieving state-of-the-art accuracy and robustness through hyperparameter-optimized meta-learning.

Our experimental results clearly demonstrate that combining the outputs of multiple base classifiers and fine-tuning them using hyperparameters leads to improvements in accuracy, precision, recall, and F1-score. These technological advances have the potential to improve email system functionality, strengthen spam defenses, and minimize resource usage. Our results validate stacking as a cornerstone technique for next-generation spam detection, bridging the gap between academic innovation and industrial practicality.

However, the current training data sourced from Kaggle and Enron datasets exhibit critical representational gaps that hinder real-world generalization. First, these datasets predominantly contain English-language spam, overlooking prevalent non-English threats such as Chinese phishing campaigns leveraging localized social engineering tactics. Second, emerging attack vectors like AI-generated emails, which mimic writing styles of specific individuals using Deepseek or similar models, are absent from the training corpus. Such omissions create domain adaptation challenges. To address this, future work will focus on integrating transformer-based classifiers for contextual analysis, applying cross-lingual transfer learning (e.g., multilingual BERT fine-tuning), and implementing adversarial training with synthetically generated attack samples, ensuring robustness against evolving threat landscapes. Leveraging these advanced models could mitigate challenges in spam detection, including adapting to changing spam strategies and reducing false positives, ultimately contributing to more resilient and effective solutions.
